# Stressful Life Events Are Related to More Negative Interpretations, but Not Under Acute Stress

**DOI:** 10.1177/00332941211014150

**Published:** 2021-05-08

**Authors:** Kathy Bélanger, Isabelle Blanchette

**Affiliations:** Département de Psychologie, Université du Québec à Trois-Rivières, Canada; Département de Psychologie, Université du Québec à Trois-Rivières, Canada

**Keywords:** Interpretation bias, stressful life events, induced acute stress, visual ambiguous stimuli, coping, executive function

## Abstract

Studies have identified deleterious effects of stress on multiple cognitive processes such as memory and attention. Little is known about the impact of stress on interpretation. We investigated how an induced acute stress and more long-term stress related to life events were associated with interpretations of ambiguous stimuli. Fifty participants answered a questionnaire indexing the number of stressful life events. A median split was used to compare those reporting few or more events. Half of participants performed an arithmetic task that induced acute stress; they were compared to a control group performing a less stressful task. We measured the interpretation of ambiguous visual stimuli, which participants had to judge as “negative” or “positive”. We found a significant interaction between the number of stressful life events and the induced acute stress on the proportion of positive interpretations. In the control group, participants reporting more stressful events produced less positive interpretations than those reporting few events. In the induced stress condition, no significant difference was found. Life events tend to influence interpretation in the absence of an acute stressor, which seems to be more influent in the short term.

## Introduction

Stress is considered the health epidemic of the 21st century by the World Health Organization (Fink, 2016). Since the seminal work of Hans Selye (1907-82), studies on stress have proliferated and exposed serious financial, emotional and physical health impacts (Fink, 2016). Global health consequences of stress range from cardiac problems, to mental illnesses and impaired cognitive performance. In this study, we focus on how stress affects cognition, more precisely how it affects the interpretation of stimuli in our environment.

Interpretation involves giving meaning to what we perceive (Wisco & Nolen-Hoeksema, 2010). It is a central component of social interactions; studies underline that individuals try to reduce uncertainty and make sense of situations in communication (Saint-Charles & Mongeau, 2009). In a laboratory context, researchers usually measure interpretation by presenting ambiguous visual or audio stimuli or ambiguous sentences or scenarios (Schoth & Liossi, 2017). Ambiguous stimuli require an interpretation from the participant as they can be considered either negative or positive (Jensen, 2005). Interpretation tasks can detect response tendencies where the participant may, for instance, have a bias towards providing more positive or more negative interpretations.

Research has shown that the way individuals interpret their environment is sensitive to their life history and psychological state (DeDora et al., 2011; Voss et al., 2008). For example, studies on people suffering from anxiety (Ogniewicz, 2012) and depression (Beck, 2008) reveal a negative interpretation bias when facing ambiguity. Anxious and depressed people tend to interpret ambiguous information, faces for example, in a negative or threatening manner (W. H. Liu et al., 2012; Richards et al., 2002). The same has been observed with individuals suffering from PTSD (Boffa, 2015; Jensen, 2005) and burnout (Bianchi et al., 2018).

Although stress is related to the previously cited disorders (Zanon et al., 2020) and is present in a daily fashion for almost everyone, no study has specifically examined the impact of non-pathological stress on interpretation. In this study, we focus on two types of stress: one that is acute (experimentally induced) and one that is related to stressful life events (more long term). We are interested in their single and combined impact on interpretation.

One way to study acute stress is by experimentally inducing stress in a laboratory. A mental arithmetic task is a well-known way of inducing stress (Campbell & Ehlert, 2012; McHugh et al., 2011; Winward et al., 2014). Acute stress increases physiological arousal - allowing to precisely monitor its progression, for example through the variations of the number of heartbeats per minute (Kudielka et al., 2004). Heartbeats per minute usually increase at the beginning of the stressful task and then decrease progressively (Mathias et al., 2004).

To our knowledge, no study has examined the link between acute stress and interpretation. Some studies have identified that stress impacts our cognitive function: for instance stress can lower scores in memory and attention tasks (Li et al., 2014; Lupien, 2015; Morelli & Burton, 2009; Starcke et al., 2016) and affect mental flexibility (Marko & Riečanský, 2018) as well as learning and decision making (Allen et al., 2014). Because stress is generally a negatively valenced state, based on the fact that other negative states lead to a negative interpretation bias, we propose that acute stress will lead to a negative interpretation bias.

Major life events such as divorce, moving or unemployment are considered stressful as they trigger a need for long-term adaptation and adjustment (Phillips et al., 2005; Vinokur & Selzer, 1975; Wethington, 2016). A common way of assessing life events is by using checklists including ratings of the desirability and recency of each event (Sarason et al., 1978). Studies have shown correlations between the number of stressful life events experienced and deleterious effects such as depression, anxiety and physical diseases (Holmes & Rahe, 1967; Phillips et al., 2015; Sharpley et al., 2004). A growing body of literature is also available on the link between life events and interpretation. For example, the occurrence of early life stress is associated with a negative interpretation bias (Williams et al., 2009). Non-specific chronic stress has also been linked to a greater negativity bias in an interpretation task, particularly in women (Braund et al., 2019). In line with this literature, we hypothesized that the experience of a greater number of stressful life events would lead to negatively biased interpretations.

Separately, studies on these two types of stress suggest that higher levels of stress should be related to a negative interpretation bias. But what about their combination? To our knowledge, there is only one study (Bélanger & Blanchette, 2020) examining whether acute stress and stressful life events produce additive or interactive effects on an interpretation task. Some studies exploring physiological stress reactions to these two stressors suggest that the combined effect may not be linear. For example, in their meta-analysis, Chida and Hamer (2008) found that participants reporting more life events presented a reduced cardiovascular reactivity to a laboratory stressor (the difference between the cardiac frequency at baseline and during the task). Another study indicated that early life adversity was associated with reduced cardiac and cortisol responses to a laboratory stressor (Lovallo et al., 2012). A study with a large sample (*n* = 585) also found a negative correlation between cardiac reactivity and the frequency of life events (Phillips et al., 2005). Altogether this shows that prior stressful experiences may not necessarily be associated with an exacerbated impact of stress, at least on physiological responses. Thus, the impact of stressful life events and acute stress may not be simply additive.

Another variable that may come to bear on the impact of stress is coping style. Coping is the constantly changing cognitive and behavioral efforts made to manage specific external and/or internal demands that are appraised as exceeding the resources of the person (Lazarus & Folkman, 1984). Weinberger et al. (1979) found that some people showed a discrepancy between levels of self-reported anxiety and of behavioral (non-verbal) anxiety in a stressful situation. They reported less stress than their body was experiencing. These individuals are called ‘repressors’ and are defined in Weinberger’s et al. (1979) theory by a low score on a trait anxiety scale and a high score on a desirability scale.

This repressive coping style has been linked to interpretation. It involves an avoidant interpretation bias - defined as a tendency to interpret ambiguous stimuli in a relatively nonthreatening fashion (Walsh et al., 2015). According to the vigilance avoidance theory (Derakshan et al., 2007), repressors tend to show a cognitive avoidance of negative stimuli, that is preceded by early vigilance, particularly with self-relevant threatening information. They reject any negative aspects of a stimulus or story (Saunders et al., 2014) and avoid retrieving threatening information from memory (Derakshan et al., 2007). Considering these previous studies, we expected that those who score high on desirability and low on trait anxiety (repressors) may present an avoidant interpretation bias and give neutral or more positive interpretations of ambiguous stimuli.

We had three objectives with this study. First, we wanted to determine if stress impacts the interpretation of ambiguous stimuli, as depression and anxiety do. We observed the single impact of acute stress and of stressful life events on the interpretation of ambiguous stimuli (1). Second, we wanted to determine whether acute stress and long-term life events have additive or interactive impacts on interpretation (2). Following the literature on physiology, we hypothesized that these two types of stress would not lead to a cumulative negative interpretation bias. Third, we wanted to assess the relative contribution of the repressive coping style, including its anxiety and desirability dimensions and their interaction, on the interpretation task (3).

## Method

### Participants

This study included 50 participants (42 females). Participants were contacted by email after they answered a recruitment ad posted on campus at the Université du Québec à Trois-Rivières. Mean age was 30 years (*SD* =13.6). Participants were equally and randomly distributed between the control and induced stress groups. Volunteers were compensated 10 dollars for their participation. This study was approved by the Ethics Committee at Université du Québec à Trois-Rivières.

### Overview of procedure

The study was conducted in a single session. Participants read and signed the consent form. Following this, the experimenter gave them more information concerning the electrocardiography procedure and participants could ask questions. They filled-out the questionnaire assessing life events. The researcher and participant then affixed the electrodes to record heart rate. The researcher explained the computer task, including the mental arithmetic task. The task began with a 3 minute-resting period to record baseline heart rate, then participants completed the other self –report questionnaires about depression, anxiety, and desirability. This was followed by the first block of mental arithmetic, the first block of the interpretation task (presenting ambiguous facial expressions), a second block of mental arithmetic and the second block of the interpretation task (presenting japanese symbols). The final block comprised a 3 minute-resting period to assess resting heart rate again.

### Material and procedure

#### Self-report questionnaires

***Life Experiences Survey (LES,*** Sarason et al., 1978 ***).*** This questionnaire contains a list of 55 potentially stressful life events (divorce, death, moving). Participants simply indicated which event happened to them, as well as the recency (in the last year, more than a year ago) and impact (very negative, negative, positive, very positive) of each relevant event. We used a median split of the total number of reported events as an independent variable to compare two groups (participants reporting fewer events, more events) in our study. Though dichotomizing a continuous measure is not recommended in some cases, we were interested in comparing both the means of the stress induction groups and those of the life events groups. This would allow us to identify any interaction effect and improve interpretability. Other studies in humanities artificially dichotomized life event measures (Kiive et al., 2017; Kuiper et al., 1986; Lovallo et al., 2012; Roy et al., 1998; Williams et al., 2009). There were similar numbers of participants in the low and high LES in the two conditions. In the control condition, there were 12 participants in the low LES score and 11 in the high. In the stress condition, there were 13 participants in the low LES score and 12 in the high. We did not test the LES for reliability in our sample as internal consistency is not expected in potential stressful life events measures: items may or may not cooccur.

***Beck Depression Inventory (BDI-II;* *Beck et al.,*** 1996 ***).*** This questionnaire includes 21 items that survey depressive symptoms experienced during the last week. Participants answered questions about sadness, changes in appetite and loss of pleasure, for example, rating each on a scale between 0 and 3. Scores range from 0 to 63. Creating authors indicate good internal consistency with an alpha of .93 and a test-retest stability also of .93. A reliability analysis in our sample indicates a Cronbach’s alpha of α =.89.

***Psychological Stress Measure (PSM-9;*** Lemyre & Tessier, 1988**).** This 9- item questionnaire evaluates the “feeling of being stressed” in the last 5 days. Psychometric details are included in this review: Gélinas et al., (2017). A reliability analysis in our sample indicates a Cronbach’s alpha of α = .84.

***Marlowe-Crowne Social Desirability Scale (MC-SDS,* *Crowne & Marlowe,*** 1960**).** This questionnaire investigates the need for social approval. High scores indicate a tendency to seek social approval and to protect self-esteem. Authors indicate test-retest correlation of .89 and internal consistency of .88 in a student sample. A reliability analysis in our sample indicates a Cronbach’s alpha of α = .33. The score to this test is used along with trait anxiety to establish coping style in our study.

***State-trait Anxiety Inventory (STAI,*** Spielberger et al., 1983 ***).*** This test measures two aspects of anxiety: state anxiety (fear sensation, temporary nervosity) and trait anxiety (stable dispositional anxiety). Scores on each scale can range from 20 to 80. Internal consistency coefficients are between .86 and .95 and test-retest value is between .65 and .75 according to the initial authors. Reliability analysis in our sample indicate Cronbach’s alpha of α = .92 (state) and α = .93 (trait). We used the trait-anxiety scale score to establish coping style in our study.

#### Experimentally induced stress

A computerized version of The Paced Auditory Serial Addition Task (PASAT-C; Lejuez et al., 2003) was used to experimentally induce stress. Originally created as a neuropsychological assessment to investigate divided and sustained attention, complaints from patients indicated that the test unintendedly induced very high levels of stress (Holdwick & Wingenfeld, 1999; Mathias et al., 2004; Tombaugh, 2006). It has often been used to induce experimental stress (Lejuez et al., 2003; McHugh et al., 2011; Winward et al., 2014). The PASAT-C is a mental arithmetic task consisting in blocks of additions. Respondents must perform the addition of two numbers mentally and answer fast. In our study, 60 black single-digit numbers were presented one by one on a white screen. The participant had to sum the last two digits shown and type the result on the keyboard (e.g: 9 and 5 = 14; 5 and 4 = 9). The answer was always between 10 and 18 to standardize response execution time. When participants provided a wrong answer or in the absence of answer, feedback was given by an irritating and sudden bomb-like sound. Additional feedback was also provided by a written score in percentage presented after each answer.

Participants performed two blocks of additions which were slightly different: the first one had a 3 –second interval between digit presentation and the second had a 2,6 - second interval. This prevented habituation and maintained the stressful effect. This task demands a high level of focus and is meant to be challenging. The mean accuracy score is expected to be about 60% for the 3-second interval block (Brooks et al., 2011). In our study, accuracy score is 83% for the first block and 88% for the second.

The control group (no induced stress) was exposed to the same sequence of single digits in each of the two blocks. However, participants in this group only had to press key “1” when they saw “1” on the screen. This happened three times (3) to keep them alert.

#### Stimuli

##### Faces

Thirty black and white pictures of ambiguous faces (15 men and 15 women) were used in the interpretation task. Pictures were taken from the Psychological Image Collection at Stirling (PICS - pics.stir.ac.uk, 2012) and from the International Affective Picture System (Lang et al., 2008). As a validation, an independent group of 10 participants was asked to categorize each picture as negative or positive. Faces that were not unanimously rated as negative or positive were considered ambiguous. During the task, participants saw one face at a time on the screen along with the question “Do you think the face is? “. The answer was a forced choice between Negative (0) or Positive (1) on the keyboard. Each face was shown twice, in a random order, totalizing 60 trials. The mean score on this task represents the proportion of positive answers and indicates the tendency to provide more positive interpretations.

##### Symbols

Thirty black and white pictures of Japanese Kanji symbols were presented. Pictures were taken from internet (“Learning Kanji”, 2016; Ray, 2016). Kanji were chosen because they are abstract visual stimuli (Huppert et al., 2003; Schoth & Liossi, 2017; Yoon & Zinbarg, 2008). During the task, participants saw one symbol at a time on the screen along with the question “Do you think the symbol is? “. Again, the answer was a forced choice between Negative (0) or Positive (1) on the keyboard. Each symbol picture was shown twice, in a random order, totalizing 60 trials. The mean score on this task represents the proportion of positive answers and indicates the tendency to provide more positive interpretations.

#### Manipulation check

##### Self-reported stress

Participants were asked to indicate their momentary perceived level of stress on a visual analogue scale between 0 which represented “not stressed at all” and 100 which represented “very stressed”. They answered by entering the corresponding number on the keyboard. They were asked four times: at the beginning of the task after the 3 minute-resting period (Time 0), then after each block of arithmetic task (Time 1 and 2) and another time at the very end after the 3 minute-recovery period (Time 3). A visual analogue scale is a fast and efficient way to assess the subjective stress level (Ali et al., 2020; Fujiwara & Okamura, 2018; J. J. Liu et al., 2020; Nakamura et al., 2020).

##### Electrocardiography

We recorded the electrical activity of the heart continuously during the whole task. Five 10-millimetres electrodes were located according to the standard 5- lead system, with four electrodes to the corners of the ribcage and one precordial (Barill, 2003). The amplifier was Powerlab (AdInstruments) and the LabChart program (AdInstruments) allowed us to record, read, extract, and analyse the data. A sampling of 1000 Hz, a record range of 20mV and filters (high pass) of 20Hz and (low pass) of 0.5Hz were used with small variations for some participants. We were especially looking at the number of beats per minute (BPM). Though recording during the whole task, four periods are of interest for the manipulation check: the last 60 seconds of the 3-minute resting period (to ensure participants were as relaxed as possible) to establish Time 0 (baseline), both 3-minute arithmetic blocks for Times 1 and 2 and the 3-minute recovery period at the very end for Time 3.

### Statistical analyses

Statistical analyses were performed in SPSS (Version 26). Preliminary data screening indicated that the data met the assumptions for t-test, ANOVA and regression analyses. Only self-reported stress scores residuals were not normally distributed because of skewness to the left. As the sample sizes of comparison groups were equal, we proceeded with the ANOVA since it is robust for normality deviations. For repeated measures ANOVAs, Mauchly’s test indicated that the assumption of sphericity had been violated, so we provided results with the Greenhouse-Geisser correction. Partial eta-squared (η2) effect size estimates were calculated for each analysis. Significance was established at the alpha level of .05.

## Results

### Descriptive analyses

Participants assigned to the control and experimental groups were similar in terms of age, mean scores on the five self-reported tests and average baseline beats per minute (see Table 1). For all dependent measures (self-reported questionnaires, beats per minute and self-reported stress score), t-test between the control group (no induced stress) and the stress induction group were non-significant showing an overall homogeneity in the sample. Only the comparison on self-reported stress between groups yielded a significant difference, described in the next page.

**Table 1.** Sample descriptives.

	Condition: no induced stress		Condition: induced stress	
	M	SD	M	SD
Age	30 years	12.8	31 years	14.6
Life Experiences Survey	12	5	13	6
Questionnaires				
BDI	10	7	11	8
MSP	35	12	37	11
STAI-Y-A	35	10	36	10
STAI-Y-B	40	12	41	12
MCSDS	18	3	19	3
Reported stress				
Time 0	24	22	30	21
Time 1*	23	21	53	24
Time 2 *	23	23	48	27
Time 3	17	19	23	19
Beats per minute BPM				
Time 0	77.37	11.4	77.66	9.8
Time 1	74.52	9.8	74.75	11.7
Time 2	75.45	9.8	76.34	9.4
Time 3	74.81	8.8	73.51	7.7

*p < .05.

### Manipulation check

#### Self-reported stress

A repeated 2 (Induced stress: control, experimental) x 4 (Time: 0, 1, 2 and 3) ANOVA indicated a significant interaction *F*(2.38,114.23) = 14.02, *p* < .001, η_p_^2^ = .23. Post-hoc comparisons revealed that the mean scores of the experimental and control conditions differed at Time 1 and 2 (see Table 1). These times correspond to the ratings given after the two arithmetic blocks. Indeed, the experimental group presented higher mean scores at both times – indicating that the stress inducing arithmetic task was successful. The scores did not differ between groups at baseline (Time 0) and recovery (Time 3). The main effect of Time *F*(2.38,114.23) = 23.44, *p* < .001, η_p_^2^ = .33 and that of Induced stress *F*(1,48) = 8.78, *p* < .01, η_p_^2^ = .16 were also significant^1^.

#### Electrocardiography (BPM)

In order to investigate if the experimentally induced stress impacted physiological responses as it did self-reported stress, we compared the mean BPM with a 2 x 4 mixed ANOVA including Induced stress (control, experimental) and Time (time 0, 1, 2, 3). The interaction was not significant, *F*(2.32,97.62) = 1.29, *p* = .28, nor was the main effect of Induced stress, *F*(1,42) = .04, *p* = .85.The main effect of time was significant *F*(2.32,97.62) = 4.02, *p* = .01, η_p_^2^ = .09, with beats per minute (BPM) being the highest at the baseline and the lowest at the end of the task. Beats per minute did not increase with the stress induction.

### Interpretation of ambiguous stimuli

#### Faces

**Figure 1.**
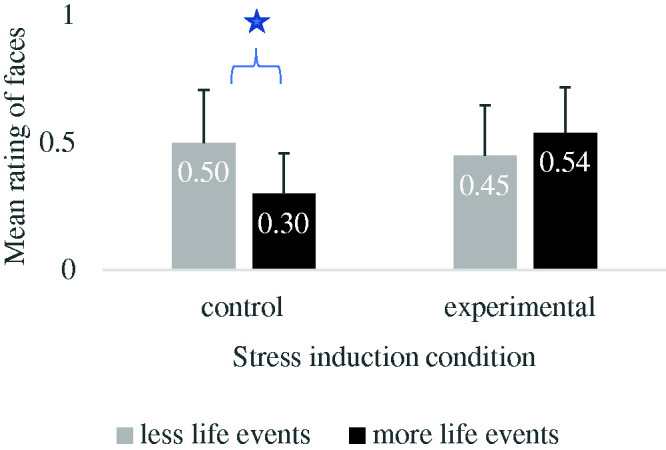
Interpretation means of the faces according to the number of life events and the condition. Error bars represent standard deviation of the mean. *p < .05.

A 2x2x3 mixed ANOVA comparing the mean interpretation of faces according to Face type (negative, ambiguous, positive), Induced stress (control, experimental) and Life events (fewer events, more events) indicated a significant interaction between Induced stress and Life events *F*(1,44) = 7.18, *p* < .01, η_p_^2^ = .14 (see Figure 1). For participants in the control condition (no induced stress), those who reported more life events interpreted the faces generally more negatively (*M* = .30; *SD* = .06) than those who reported fewer life events (*M* = .50; *SD* = .06) *F*(1,21) = 6.53, *p* < .05, η_p_^2^ = .24. In the induced stress condition, no significant difference was found; participants reporting more life events did not interpret faces more negatively *F*(1,23) = 1.46, *p* = .24.

The main effect of Face type was significant *F*(1.72,75.80) = 200.96, *p* < .001, η_p_^2^ = .82. Positive faces were interpreted more positively (*M* = .67, *SD* = .23) than the ambiguous faces, and the ambiguous faces more positively (*M* = .46, *SD* = .22) than the negative faces (*M* = .22, *SD* = .22). The main effects of Induced stress *F*(1,44) = 2.70, *p* = .11 and of Life events *F*(1,44) = 1.01, *p* = .32 were not significant.

#### Symbols

**Figure 2.**
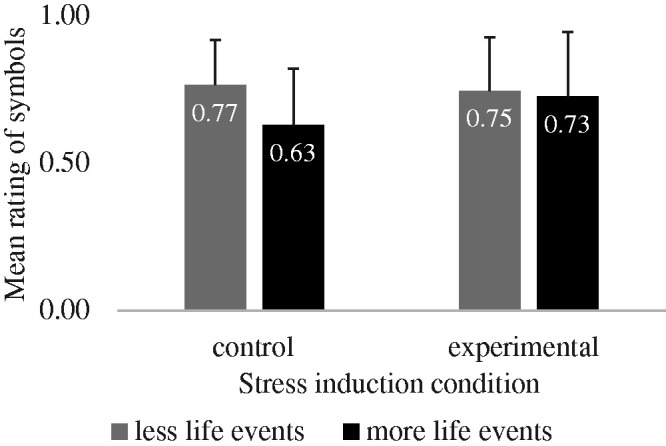
Interpretation means of the kanji according to number of life events and the condition. Error bars represent standard deviation of the mean.

A 2x2 mixed ANOVA comparing the interpretation of Kanji symbols according to Induced stress (control, experimental) and Life events (few events, more events) did not show a significant interaction *F*(1,43) = 1.13, *p* = .29, η_p_^2^ = .03 nor any main effect (all *p*s > .16). However, the descriptive pattern of means mirrored the one of the face stimuli; in the control group, participants who reported more stressful events interpreted the symbols more negatively than those who reported few stressful events. In the experimental group, the interpretation of kanji symbols was more similar across groups reporting different numbers of life events (see Figure 2). It is worth noting that kanji were rated more positively than the faces in general (*M* = .72, *SD* = .19 and *M* = .45, *SD* = .20 respectively).

### Moderation effect

A hierarchical multiple regression was used to examine the contribution of trait anxiety and desirability to the bias of the faces. The criterion variable was the proportion of positive interpretations. We first tested a model including age, induced stress, the number of life events reported as a continuous variable and the interaction between induced stress and life events (the interaction was calculated by standardizing each variable scores and using the cross-product) to establish a reference model of contributions. In this first model, only age was a significant predictor and the number of life events was a marginally significant predictor (see Table 2).

**Table 2.** Multiple regression of independant variables on mean face interpretations.

Variable	B	*SE* B	Beta	*t*
A				
Age	.005	.002	.352	2.53*
Number of life events	−.011	.006	−.301	−1.81
Induced stress	.019	.069	.047	.28
Life Events × Induced Stress	.133	.095	.287	1.40
Constant	.382			
B				
Age	.007	.002	.443	3.27**
Number of life events	−.008	.005	−.215	−1.60
Desirability scale	.024	.009	.361	2.56*
Trait Anxiety Scale	−.002	.002	−.119	−.85
Desirability × Trait Anxiety	−.025	.025	−.132	−.99
Constant	.005			

Note: Fit for model *R*^2^ = 0.24, *F*(4,47) = 3.38, *p* < .05 (a). Fit for model *R*^2^ = 0.29, *F*(5,47) = 3.36, *p* < .05 (b).

*p < .05. **p < .01.

In the second model, we retained the best contributing variables (age, life events) and we tested if coping style explained additional variance by including trait anxiety scores, desirability scores and their interaction (the interaction was calculated by standardizing each variable score and using the cross-product). This second regression model indicated that age significantly predicted mean ratings of the faces as did the desirability score. Life events, trait anxiety and the interaction term failed to predict interpretation (see Table 2).

## Discussion

Our study sheds light on the influence of stress on the interpretation of the environment. We were interested in distinguishing the single and combined effects of an induced acute stress and more long-term stress resulting from life events on the interpretation of ambiguous visual stimuli. Based on physiological studies, we hypothesized that the combination of life events and an experimentally induced stress would not lead to more negative interpretations of ambiguous stimuli. A significant interaction was found between the number of stressful life events and the experimentally induced stress condition on the interpretation of faces. When no acute stress was present, participants reporting a greater number of stressful life events produced more negative interpretations than those reporting fewer stressful life events. However, when an acute stress was experimentally induced, participants produced similar interpretations of the ambiguous stimuli regardless of the number of reported stressful life events.

This interactive effect reveals that the combination of both types of stressors did not exacerbate a negative interpretation bias. In the absence of acute stress, the effect of life events is comparable to the one observed with depression and anxiety disorders on interpretation (Beck, 2008; Ogniewicz, 2012). However, in the acute stress condition, the induction seems to have buffered participants from the influence of life events (Cibrian-Llanderal et al., 2018; Klein & Boals, 2001; Shields et al., 2016). This may result from the fact that the immediate stressor recruits available cognitive resources and makes judgments less permeable to longer-term influences. We ensured that the stress induction was successful with the self-reported stress scores and the average heartbeats per minute. On the visual analogue scale, participants under the stress induction reported that they felt more stressed than the participants in the control condition (see Table 1). We could not validate this impact with the heartrate measure (see Table 1), contrary to previous studies (Phillips et al., 2005; Starcke et al., 2016). Other studies have reported similar lack of effect of the PASAT-C on physiological indices when interval rates were over 2,4 seconds – we used 3.0- and 2.6-seconds intervals (Tanosoto et al., 2015). Moreover, in our study, participants showed the highest heartrate at the start of the experimental session, suggesting they negatively anticipated the task. Our study recruited participants from the general population, aged 18 to 65 years old, with the objective of varying from the typical university student sample. This diversity may favor the unfamiliarity with experimental studies and explain the elevated HR at the beginning of the session instead of during the session.

Life events are loaded with emotional content and they can trigger stressor-related intrusive thoughts. These thoughts may interfere with optimal executive functions during a task – in our case interpretation of ambiguous stimuli (Cann et al., 2011; Dougall et al., 2012). In the control group, participants reporting more life events might have experienced intrusions relation with the cognitive load of life events. In our study, the cognitive load of stressful life events could explain the link between life events and more negative rating of the ambiguous faces. We suspect this effect to be overridden with the co-occurrence of an acute stress that requires focus (Klein & Boals, 2001). This suggests that stress is not simply linearly related to negative interpretations, as are anxiety disorders and depression. Life events tend to influence behavior in the absence of an acute stressor, which seems to be more influent in the short term.

We also wanted to assess the contribution of repressive coping style in interpretation bias. We measured this using the interaction of trait anxiety and desirability scores in accordance with Weinberger’s et al. (1979) theory. We could have expected an avoidant bias, reflected in neutral or more positive interpretations from participants scoring low on anxiety and high in desirability scales. Through multiple regression, the interaction term was not significant, indicating that a repressive coping style was not related to an interpretation bias. Previous work on repressive coping style provide hints to explain this result. The vigilance-avoidance theory (Derakshan et al., 2007) pinpoints a temporal framework where repressors demonstrate vigilance at an early stage of a task and avoidance later. Avoidance stage is characterised by the use of avoidant cognitive bias. In our study, participants had to provide an answer with a forced choice between ‘negative’ and ‘positive’. Repressors may have deployed avoidant strategies, resulting in more neutral answers, not necessarily overly positive interpretations (Myers, 2010). To explore this result a step further, other studies highlight the fact that the avoidant bias is triggered by specific situations and stimuli such as threatening stimuli as well as self-relevant threat and social situations (Derakshan et al., 2007; Walsh et al., 2015). Our visual ambiguous stimuli were neutral and may not have triggered a threat perception to elicit a defensive reaction (avoidant bias). Also, some authors question the contemporary validity of the desirability scale (it has been developed in 1960) and therefore its current use in accurately identifying a repressive coping style (Walsh et al., 2015).

The moderation analysis showed no contribution of trait anxiety scale score in the interpretation. This finding is not congruent with the extensive work on anxiety and interpretation, generally showing a negative interpretation bias (Blanchette & Richards, 2010; Huppert et al., 2003). A study examining trait anxiety and its relation to interpretation bias found that low trait anxiety was related to an avoidant bias, using a cutoff score of 44 on the trait anxiety scale (Walsh et al., 2015). In our sample, mean score for this scale was 40 (*SD* = 11) and it could suggest a similar tendency of avoidant interpretations. Also, anxiety-related biases may not be triggered by all types of stimuli. Some studies suggest that they are mostly evident for situations related to social anxiety (Constans et al., 1999; Walsh et al., 2015) which is not what was used in our study. We found that the desirability scale score was a significant predictor of the interpretation of ambiguous stimuli, with participants higher on desirability exhibiting more positive interpretations. The desirability scale is intended to detect a tendency to show defensiveness and protection of self-esteem, to maintain a positive image of the self, as well as inhibition of affect and avoid introspection (Crowne & Marlowe, 1960). In our study, participants with high desirability scores produced more positive ratings of the ambiguous faces, possibly in an attempt to waive negativity (Furnham et al., 2003). Lastly, our regression results also support the age-related positive interpretation bias reported in other studies - being older was associated with more positive ratings of the faces in general (Reed et al., 2014). The adjusted R square values of our models are considered within an acceptable range in the social sciences (Xiao & Hu, 2019). Also, our intention aimed to explore the relationship between variables, not to list the multiple causes to interpretation, therefore limiting the predictability power (Moksony, 1990).

Overall, in our study, ambiguous faces were more useful visual stimuli to study interpretation than kanji were; these did not allow us to detect any differences according to the number of life events or the stress condition. An explanation for this absence of result may lie in the fact that for Westerners, Japanese symbols are quite exotic and mostly related to movies and food. So, while kanji are ambiguous visual stimuli, their perception may be positively biased, masking effects of stable or temporary differences in affective state.

Our study has some limitations. First, the stress induction did not produce the expected effect on heartrate. In our sample, participants had the highest heart rate at the baseline level and not during the PASAT-C. Also, the use of a median split with the life events measure limits the generalization of our findings. It is not possible to predict tendencies in interpretation at higher levels of life events exposition.

In this paper, we wanted to examine the impact of non-pathological stress on interpretation. We found that when no acute stress was present, participants reporting a greater number of stressful life events produced more negative interpretations of ambiguous faces. In the presence of acute stress, all interpretations were similar regardless of the number of life events. Also, we could not relate the repressive coping style to an interpretation bias. We recommend investigating negative interpretation bias and stressful life events as they are related to some health and cognitive hazard and account for a fertile ground in mental illness (Phillips et al., 2015; Sandi, 2013; Schoth & Liossi, 2017). Dealing with stressful life events may shape our perception of the world and influence our global health as well.

## Note

1 A sensitivity power analysis was performed with G*Power software. An ANOVA with 50 participants across two groups would be sensitive to effects of η_p_^2^= .04 with 80% power (alpha = .05).

## Notes

**Author Biographies:** **Kathy Bélanger:** is a doctoral student at Université du Québec à Trois-Rivières. She conducts research on the impact of stress on the interpretation of ambiguous stimuli. **Isabelle Blanchette:** is a professor of Psychology at Université Laval. She received her PhD from McGill University and did a post-doc at Birkbeck College, University of London. Her research interests focus on the link between cognition and emotions.
